# Deliberation and Procedural Automation on a Two-Step Task for Rats

**DOI:** 10.3389/fnint.2018.00030

**Published:** 2018-08-03

**Authors:** Brendan M. Hasz, A. David Redish

**Affiliations:** ^1^Graduate Program in Neuroscience, University of Minnesota Twin Cities Minneapolis, MN, United States; ^2^Department of Neuroscience, University of Minnesota Twin Cities Minneapolis, MN, United States

**Keywords:** decision-making, reinforcement learning, model-based, model-free, vicarious trial and error, path stereotypy

## Abstract

Current theories suggest that decision-making arises from multiple, competing action-selection systems. Rodent studies dissociate deliberation and procedural behavior, and find a transition from procedural to deliberative behavior with experience. However, it remains unknown how this transition from deliberative to procedural control evolves within single trials, or within blocks of repeated choices. We adapted for rats a two-step task which has been used to dissociate model-based from model-free decisions in humans. We found that amixture ofmodel-based andmodel-free algorithms was more likely to explain rat choice strategies on the task than either model-based or model-free algorithms alone. This task contained two choices per trial, which provides a more complex and non-discrete per-trial choice structure. This task structure enabled us to evaluate how deliberative and procedural behavior evolved within-trial and within blocks of repeated choice sequences. We found that vicarious trial and error (VTE), a behavioral correlate of deliberation in rodents, was correlated between the two choice points on a given lap. We also found that behavioral stereotypy, a correlate of procedural automation, increased with the number of repeated choices. While VTE at the first choice point decreased with the number of repeated choices, VTE at the second choice point did not, and only increased after unexpected transitions within the task. This suggests that deliberation at the beginning of trialsmay correspond to changes in choice patterns, while mid-trial deliberation may correspond to an interruption of a procedural process.

## Introduction

It has long been known that multiple systems within the brain contribute to making decisions (O'Keefe and Nadel, 1978; Adams and Dickinson, 1981; Sloman, 1996; Dayan and Balleine, 2002; Lieberman, 2003; Loewenstein and O'Donoghue, 2004; Balleine et al., 2008; van der Meer et al., 2012; Dolan and Dayan, 2013; Redish, 2013). Studies of rat navigation through spatial mazes have revealed a dichotomy between deliberative behavior or “place strategies,” and procedural behavior or “response strategies” (Muenzinger and Gentry, 1931; Tolman, 1939; O'Keefe and Nadel, 1978; Packard and McGaugh, 1996; Jog et al., 1999; Redish, 1999; Gardner et al., 2013; Schmidt et al., 2013; Redish, 2016). This body of work finds that while rodents display deliberative behavior with limited training on a task, experience with a task leads to a transition toward procedurally-driven behavior. However, such tasks usually involve only a single simple choice (though see Gardner et al., 2013), making it difficult to evaluate how any transition from deliberative to procedural control may evolve over the course of single laps. Furthermore, the transition from deliberative to procedural behavior is primarily investigated either in terms of the number of training sessions, or the number of laps within a session. If procedural automation increases as a function of experience, then this procedural control should increase specifically with the number of repeated choices on a task.

Procedural automation in rats during navigation tasks has traditionally been identified by stereotyped behavior, while deliberation has been identified by variable behavior at decision points, such as vicarious trial and error (VTE). Vicarious trial and error is a behavior where rats pause at choice points of a maze, and look back and forth down each path as if deliberating over which path to take (Muenzinger and Gentry, 1931; Tolman, 1939; Redish, 2016). VTE is thought to correspond to an internal deliberative process (Redish, 2016), and has been found to co-occur with nonlocal representation by hippocampal place cells (Johnson and Redish, 2007; van der Meer et al., 2010). During procedural behavior, rats do not display VTE, and their paths through the choice points are highly stereotyped (Packard and McGaugh, 1996; Jog et al., 1999; Schmitzer-Torbert and Redish, 2002; van der Meer et al., 2012; Smith and Graybiel, 2013; Schmidt et al., 2013). The procedural system generating this stereotyped behavior is hypothesized to employ a model-free learning algorithm (O'Keefe and Nadel, 1978; Jog et al., 1999; Yin and Knowlton, 2004; Frank, 2011; Redish, 2016). Animals usually display deliberative behavior early in training, which transitions to more stereotyped behavior with experience on a given task (Packard and McGaugh, 1996; Redish, 1999; Gardner et al., 2013; Schmidt et al., 2013).

However, deliberative and procedural behavior are usually evaluated on a per-trial basis, precluding any analysis of how these behaviors might change over the course of single trials. Therefore, it is unknown whether animals deliberate over single choices independently, whether they enter deliberative or non-deliberative modes over the course of entire multi-choice trials, or whether deliberation at the initiation of a trial instigates procedural control for the remainder of the trial. Furthermore, the transition from deliberative to procedural control is measured as a function of trial within a session, or session within a training schedule. If procedural automation increases with an animal's experience with a specific action chain, then behavioral stereotypy should increase not only as a function of trial or session, but with the number of specific actions or choices that the animal has performed.

There are specific hypotheses as to the algorithms within the brain which drive procedural automation and deliberation. The procedural system is hypothesized to be driven by a “model-free” neural mechanism, in that it does not actually use a model of the world to make decisions, but stores only the expected value of taking certain actions in given states (Schultz et al., 1997; Sutton and Barto, 1998; Jog et al., 1999; Swanson, 2000; Yin and Knowlton, 2004; Niv et al., 2006; Calabresi et al., 2007; Frank, 2011). The procedural system makes decisions quickly, but these decisions are inflexible once learned (Niv et al., 2006; Johnson et al., 2007; Keramati et al., 2011; van der Meer et al., 2012). On the other hand, the deliberative system has been hypothesized to use an internal model of the world to evaluate the outcomes of potential actions, a “model-based” neural mechanism (Tolman, 1939; Doll et al., 2012; van der Meer et al., 2012; Daw and Dayan, 2014; Redish, 2016). In addition to learning the relationships between state-action pairs and reward, the internal model learns the relations between states, and that knowledge can be integrated on-line to make more optimal decisions even in novel situations (Adams and Dickinson, 1981; van der Meer et al., 2012). However, deliberation requires more time for action selection than the procedural system because it requires the simulation of an internal model (Keramati et al., 2011), which is thought to correspond to imagination of future goals (Johnson and Redish, 2007; Simon and Daw, 2011; Doll et al., 2015; Brown et al., 2016).

While procedural and deliberative behavior has been extensively studied in rodents, the model-based/model-free dichotomy has been evaluated mostly in humans (though see Miller et al., 2017). Instead of distinguishing behavior types, this literature employs tasks which differentiate specific decisions based on the apparent presence of knowledge about relations between states, information which only the model-based system stores (Daw et al., 2011; Doll et al., 2012). Consistent with a transition from deliberative to procedural control, when a mixture of model-based and model-free algorithms are used to model human choice strategies, much of this work finds that such a hybrid algorithm explains decisions better than either model-based or model-free algorithms alone (Gläscher et al., 2010; Daw et al., 2011; Gillan et al., 2011; Wunderlich et al., 2012; Eppinger et al., 2013; Otto et al., 2013a,b; Skatova et al., 2013; Gillan et al., 2014; Schad et al., 2014; Sebold et al., 2014; Deserno et al., 2015; Gillan et al., 2015; Otto et al., 2015; Radenbach et al., 2015; Sharp et al., 2015; Voon et al., 2015; Decker et al., 2016; Doll et al., 2016).

A task often used to dissociate model-based from model-free choice in the human literature is a choice task which has two stages, that is, two choices per trial (Daw et al., 2011). Studies using this task have uncovered choice behavior consistent with independent influences of model-based and model-free learning algorithms (Gläscher et al., 2010; Daw et al., 2011; Gillan et al., 2011; Wunderlich et al., 2012; Eppinger et al., 2013; Otto et al., 2013a,b; Skatova et al., 2013; Gillan et al., 2014; Schad et al., 2014; Sebold et al., 2014; Deserno et al., 2015; Gillan et al., 2015; Otto et al., 2015; Radenbach et al., 2015; Sharp et al., 2015; Voon et al., 2015; Doll et al., 2016; Decker et al., 2016). To investigate the extent to which rodent choices are influenced by model-free and model-based processes, and to evaluate procedural and deliberative behavior on a task with multiple choices per trial, we adapted this two-step task for rats. The two-step task has recently been adapted for rats in a different way by Miller et al. (2017). However, we use a spatial maze-based task, which enables us to measure behavioral markers of deliberation and procedural automation, such as VTE and behavioral stereotypy. By analyzing both the behavior and choices of rats on this task, we were able to determine the extent to which rats used choice strategies which were model-based, model-free, or a mix thereof, and determine how procedural and deliberative behavior evolved within laps and over the course of blocks of repeated choice sequences.

**Figure 1:**
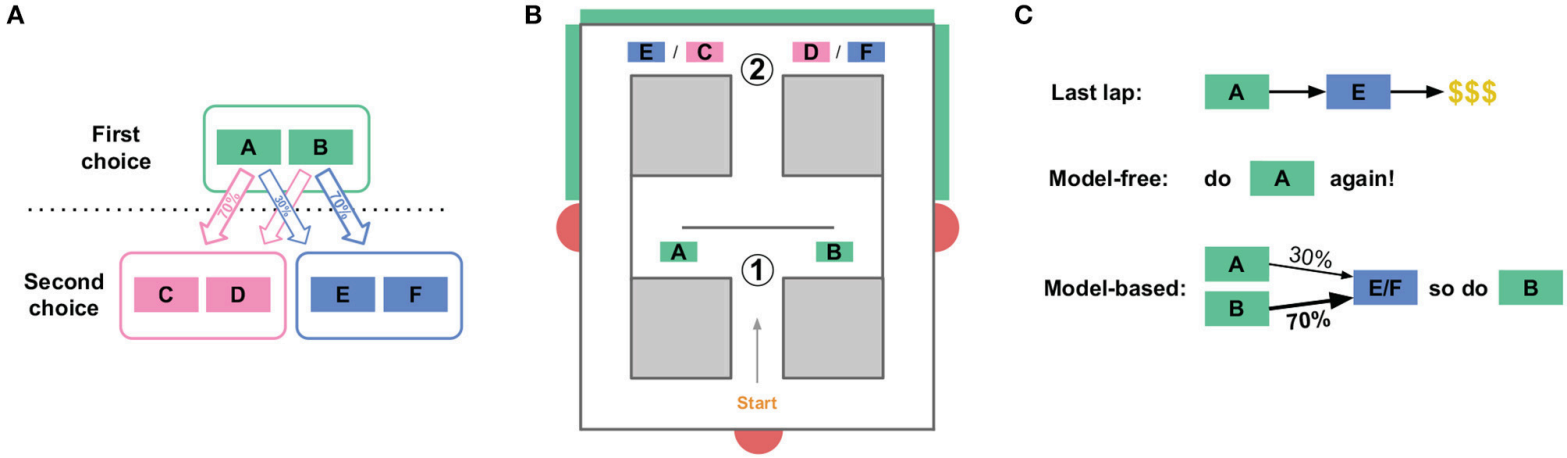
The two-step task. **(A)** State structure of the task. A first choice between two options leads probabilistically to one of two second-stage choices. Each of the four second-stage choices have some cost of reward associated with them, and those costs change over the course of the session. **(B)** The spatial version of the two-step task for rats. An initial Left/Right choice point (labeled “1,” corresponding to the first choice in **A**), leads to a second-stage choice (labeled “2”). Which of the two second stage choices is currently presented is indicated by an audio cue, and by a visual cue on monitors (green boxes on outside of maze). Rats then wait some amount of time before receiving food reward at feeder sites (red semicircles). **(C)** This task dissociates model-based from model-free choices. When an agent receives reward after a rare transition, the model-free system is more likely to repeat the first-stage choice which lead to that reward, while the model-based system is more likely to take the opposite first-stage action on the next lap.

The human two-step task (Daw et al., 2011, see Figure 1A) consists of a sequence of two choices: C1 (choosing between A vs. B) and then C2 (choosing between C vs. D) or C3 (choosing between E vs. F). Choosing option A in C1 usually but not always leads to choice C2, while choosing option B in C1 usually leads to choice C3. Choosing C vs. D (in C2) or E vs. F (in C3) leads to probabilistically-delivered reward, with different probabilities at C, D, E, and F. The probabilities change slowly over time, so the subject is constantly trying to find the best option and should not simply settle on one option, but can use observations of reward as a signal that the option is a good one to return to (at least for a while).

This two-step task is able to dissociate model-based from model-free decisions because it creates conditions where the two decision-making algorithms make different choices, mostly on laps following a rare transition (e.g., choosing A at C1 leads to C3, a choice between E and F). This is because the model-based algorithm stores information about the relation between states (specifically, the transition probabilities), while the model-free algorithm does not store information about relations between states (and so does not use the transition probabilities for valuation).

To illustrate this difference, suppose a subject chooses A at C1, experiences a rare transition and is presented with C3 (a choice between E and F), chooses E at the second choice, and receives a large reward (Figure 1C). A model-free agent would be more likely to repeat the choice at C1 (choice A), because model-free learning algorithms reinforce actions which have led to reward in the past, without taking into account relations between states. However, the world model of the model-based algorithm stores relations between states, and so has access to the fact that choosing B at C1 is more likely to lead to the C3 choice, where E can then be chosen. Therefore, the model-based algorithm would be more likely to choose B at C1 in this scenario, while the model-free algorithm would be more likely to choose A. In general on this task, model-based and model-free agents value the two choices at C1 slightly differently.

Our version of the two-step task for rats was a spatial maze with two left/right choices, which corresponded to the two choice stages in the human task (Figure 1B). The second choice (C2/C3) was the same physical location for both the C/D and E/F choices, but an audiovisual cue at the second choice point informed animals whether they were in the C2 or C3 context. Choosing left (A) at the first choice led to C2 80% of the time, and to C3 20% of the time. Like the human task, those probabilities were reversed after choosing right (B) at the first choice point. After choosing left (C or E) or right (D or F) at the second choice point, rats were rewarded with food pellets. While the cost of reward in the human task was the probability of receiving a reward at all, we used delay to food delivery as the cost: high delay to food delivery corresponded to high cost rewards, while low delays corresponded to low cost rewards. The sessions were limited to 1 h, and the rats earned their daily food intake by running the task, so they were motivated to seek food rewards with low delays. Like the human task, these delays varied between C, D, E, and F. The delays were initialized randomly between 1 and 30 s, and changed slowly over the course of a session according to a Gaussian random walk with a standard deviation of 1s/lap. There were return corridors from the reward offer sites to the start of the maze, and rats ran laps freely for 45 min per session.

## Results

### Rats displayed a mix of model-based and model-free decision making

**Figure 2:**
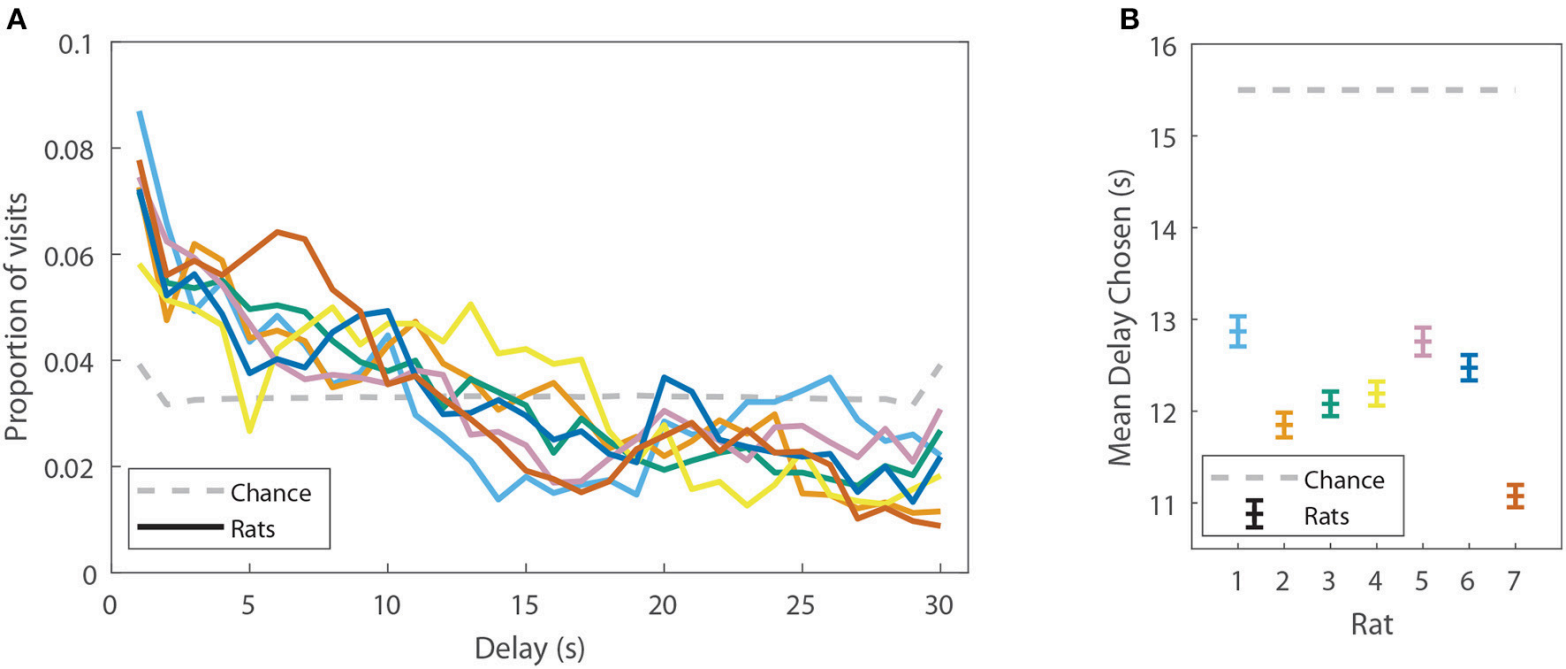
Rats display a preference for low-delay feeders on the spatial two-step task. **(A)** The proportion of delays experienced by the rats (colored solid lines, each line is one rat), as compared to the proportions of delays which would be expected by visiting feeders randomly. **(B)** The mean delay experienced by the rats (±SEM) as compared to the mean delay which would be expected by visiting feeders randomly (generated by a model-free simulation run with learning rates at 0). Delays have been aggregated over all sessions from a given rat.

Rat behavior on the spatial two-step task was collected from 7 rats for at least 48 sessions each (357 sessions in total, Table 1A). Rats ran an average of 74.2 ± 19.6 laps per session (Table 1B). Not surprisingly, rats preferred reward offers with a low delay to food delivery (Figure 2). We ran simulations of agents which made random choices on the two-step task to determine the delays which would be expected by visiting feeders randomly. Mean delays experienced by the rats were significantly less than the mean delay experienced by the random-choice simulations (two-sided Wilcoxon signed rank test, *N*_*rats*_ = 7, *p* = 0.0156, rat delays were 3.31 s lower on average than simulation delays). This indicates that rats were able to learn the task, by making decisions which led to lower-delay outcomes.

**Table 1:** **(A)** The number of sessions for each rat, and **(B)** the total number of laps each rat ran.

**(A) Sessions per Rat**	
**Rat**	**Number of sessions**
1	48
2	50
3	50
4	50
5	53
6	53
7	53
Total	357
**(B) Laps per Rat**	
**Rat**	**Number of laps**
1	3,313
2	3,602
3	4,079
4	3,610
5	3,594
6	3,805
7	4,478
Total	26,481

**Figure 3:**
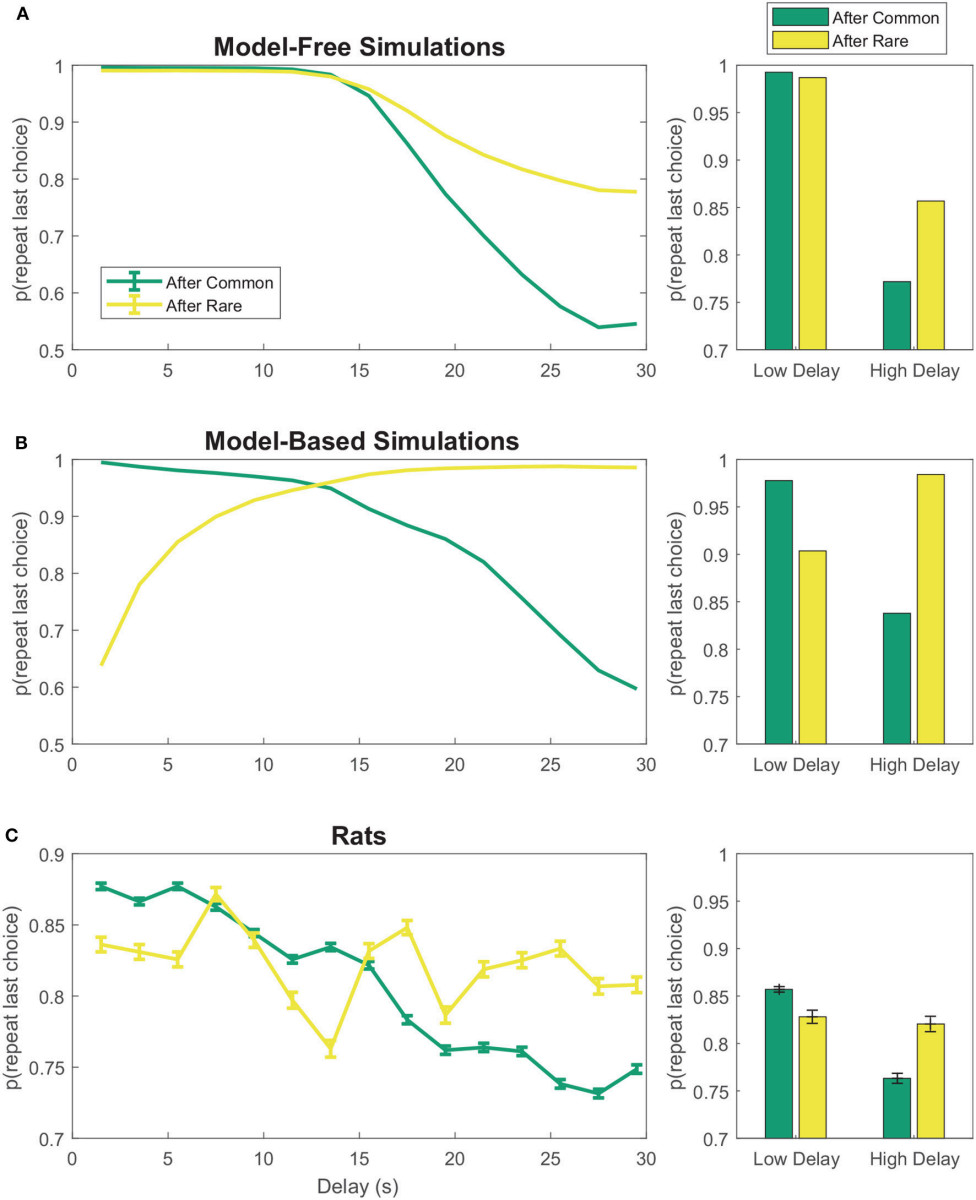
First-stage choice repetition by delay for **(A)** model-free and **(B)** model-based reinforcement learning simulations. Data has been aggregated over simulated sessions. Error bars were omitted from **(A,B)** because SEM of the simulations was negligible. **(C)** Rats show features of both model-based and model-free behavior. Data has been aggregated over rats and sessions. Error bars show SEM with *N* = the total number of laps with a given delay. Delays were binned into 2 s bins.

On the two-step task, model-free agents are more likely to repeat first-stage choices which led to low-delay (low-cost) rewards than those which led to high-delay (high-cost) rewards, even if this reward occurred after a rare transition (Figure 3A). However, model-based agents show the opposite pattern after rare transitions—that is, they are less likely to repeat first-stage choices which led to low-cost rewards than those which led to high-cost rewards after rare transitions (Figure 3B). The rats appeared neither purely model-based nor purely model-free, suggesting a mix of model-based and model-free behavior (Figure 3C), consistent with behavior seen in human subjects (Gläscher et al., 2010; Daw et al., 2011).

To more rigorously evaluate model-based or model-free influences on rat choices, we fit model-based and model-free algorithms to rat choices on the two-step task using Stan, in order to perform Bayesian inference and compare models (Kruschke, 2014; Carpenter et al., 2017). We also considered a constant-weight hybrid algorithm where choices were made according to some fixed weight (a free parameter) between model-based and model-free influence. We used Deviance Information Criterion (DIC) scores to select the most likely of these three algorithms (Spiegelhalter et al., 2002). Differences in DIC scores >7 suggest the algorithm with the higher DIC score has “considerably less support” (Spiegelhalter et al., 2002) than the algorithm with the lower DIC score.

The purely model-based algorithm was more likely than the purely model-free algorithm to explain rat choices on the two-step task (DIC score difference of 94, Tables 2A,B,D). However, the constant-weight hybrid algorithm was more likely than the purely model-based algorithm to explain rat choices on the two-step task (DIC score difference of 69, Tables 2C,D). The fact that the constant-weight hybrid algorithm had a far lower DIC score suggests that rat choices on the two-step task were driven by some combination of model-based and model-free decision making, and were not driven by either the model-based or model-free system alone. This is consistent with many human studies which find that human choices on the two-step task display both model-based and model-free influences (Gläscher et al., 2010; Daw et al., 2011; Wunderlich et al., 2012; Otto et al., 2013a,b; Doll et al., 2016).

**Table 2:** Reinforcement learning algorithm fit parameters and DIC scores.

**Parameter**	**MAP**		**Mean**		**Std**
**(A) MODEL-FREE**					
α_1_	0.0710		0.0739		0.0120
α_2_	0.00165		0.00170		0.000551
*β*_1_	3.44		3.73		1.20
*β*_2_	3.64		3.93		1.28
*p*	0.380		0.387		0.120
λ	0.00200		0.00171		0.00140
DIC score:	51,515		Log Post.:		−25,832
**(B) MODEL-BASED**					
α_2_	0.000933		0.000920		0.000240
*β*_1_	7.29		7.87		1.98
*β*_2_	6.39		6.90		1.74
*p*	0.177		0.174		0.0451
DIC score:	51,421		Log Post.:		−25,741
**(C) CONSTANT WEIGHT**					
α_1_	0.0371		0.0360		0.0196
α_2_	0.00121		0.00129		0.000360
*β*_1_	6.16		6.55		1.84
*β*_2_	4.96		5.01		1.38
*p*	0.207		0.211		0.0593
λ	0.00144		0.00190		0.00207
*w*	0.675		0.647		0.0795
DIC score:	51,352		Log Post.:		−25,735
**(D) Relative dic scores**					
**Model**	**Constant weight**	<	**Model based**	<	**Model free**
DIC difference	(most likely)	69		94	(least likely)

*The constant-weight algorithm explains rat choices better than the purely model-free or purely model-based algorithms. α_1_ is the learning rate parameter for the first choice point, and α_2_ is the learning rate for the second choice point. *β*_1_ is the inverse temperature parameter (controls how random or exploratory choices are—lower values yield more random choices) for the first choice point, and *β*_2_ is the inverse temperature parameter for the second choice point. The p parameter is a perseveration parameter which captures how likely rats were to repeat choices from the previous lap regardless of learning (larger values indicate a higher propensity for choice repetition). The λ parameter is the eligibility trace parameter, which controls how quickly value information from the second stage is propagated back to the first stage valuation in the model-free learner. The w parameter in the constant-weight model controls the weighting between model-based and model-free influence (with w = 0 the algorithm is purely model-free, and with w = 1 the algorithm is purely model-based). MAP, maximum a posteriori parameter estimate; Mean, mean of the MCMC samples; Std, standard deviation of the MCMC samples; DIC score, Deviance information criterion; models with lower DIC are more likely; DIC differences > 7 suggest the algorithm with the higher DIC has “considerably less support” (Spiegelhalter et al., 2002)*.

### Rats showed behavioral correlates of deliberation and procedural learning on the two-step task

**Figure 4:**
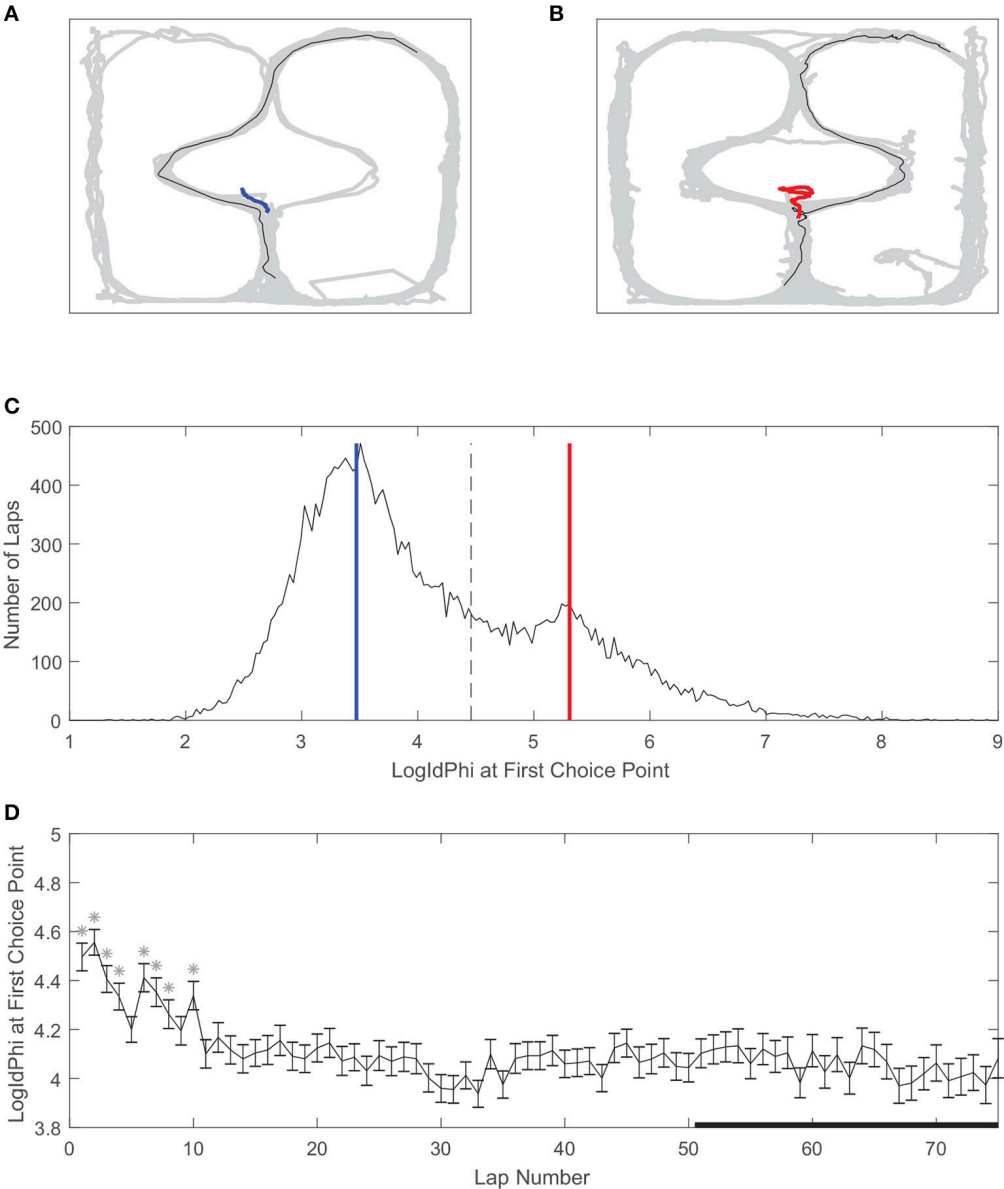
Vicarious trial and error (VTE) at the first choice point. **(A)** An example of a pass through the first choice point without VTE, and **(B)** an example of VTE at the first choice point. Gray line is rat body position over the whole session, black line is rat body position on example lap, and red or blue lines are rat head position at the first choice point on the example lap. **(C)** Distribution of LogIdPhi values at the first choice point over all laps, sessions, and rats. Blue line corresponds to LogIdPhi value at the first choice point in the example lap shown in **A**, and the red line to the example lap shown in **B**. Dashed line is the VTE/non-VTE threshold (see section Methods). **(D)** LogIdPhi over the course of a session. Error bars indicate SEM. Stars indicate laps for which LogIdPhi was significantly greater than that of laps 51 and greater. Data has been aggregated over rats (*N* = 357, the total number of sessions). Error bars show SEM.

Vicarious trial and error is a behavioral correlate of deliberation in rats (Redish, 2016). We used LogIdPhi, a measure of pausing and head-turning, to measure VTE (see section Methods). We found that on our spatial two-step task, rats displayed varying levels of LogIdPhi at the first choice point (Figure 4). There was a clear bimodal distribution of LogIdPhi at the first choice point, where one peak with lesser LogIdPhi values corresponded to laps where no VTE occurred (Figures 4A,C) and the other peak with greater LogIdPhi values corresponded to laps where VTE occurred (Figures 4B,C). The amount of VTE was greater at the beginning of a session. When comparing each lap to laps > 50, there was significantly more VTE at the first choice point for 8 of the first 10 laps. However, there was not significantly more VTE on laps 10–50 than on laps > 50 (Figure 4D, Wilcoxon rank sum test, Bonferroni corrected for multiple comparisons, with pre-correction threshold of *p* < 0.05).

**Figure 5:**
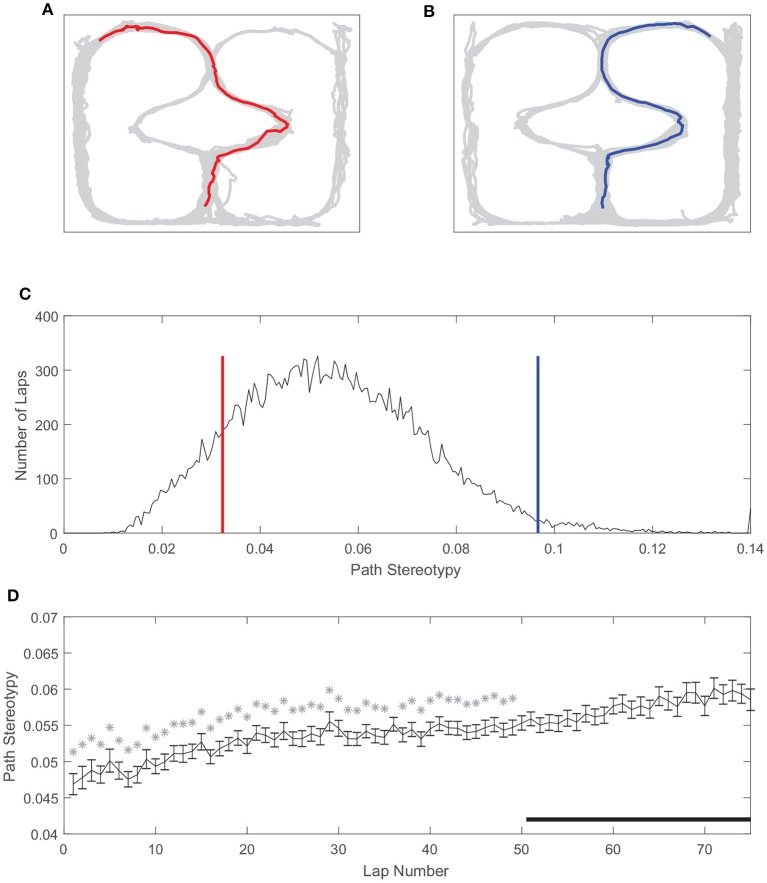
Path stereotypy on the spatial two-step task. **(A)** An irregular, non-stereotyped path, and **(B)** an example of a highly stereotyped path. The gray line is rat body position over the whole session, and colored lines are the rat body position on the example lap. **(C)** Distribution of path stereotypy over all laps, sessions, and rats. Red line corresponds to the log deviation value of the example lap shown in **(A)**, blue line to the example lap shown in **(B)**. **(D)** Path stereotypy over the course of a session. Stars indicate laps for which path stereotypy was significantly less than that of laps 51 and greater. Data has been aggregated over rats (*N* = 357, the total number of sessions). Error bars show SEM.

Similarly, path stereotypy is a behavioral correlate of procedural decision-making (Packard and McGaugh, 1996; Jog et al., 1999; Schmitzer-Torbert and Redish, 2002; van der Meer et al., 2012; Schmidt et al., 2013; Smith and Graybiel, 2013). We used the inverse of the deviation from the average path to measure path stereotypy (such that larger values correspond to greater behavioral stereotypy, see section Methods). The stereotypy of rats' paths also varied on our task (Figure 5). Unlike VTE, there was a unimodal distribution of path stereotypy, where some laps were less stereotyped (Figures 5A,C) and other laps were more stereotyped (Figures 5B,C). Also unlike VTE, path stereotypy increased steadily over the course of a session, with 48 of the first 50 laps being significantly less stereotyped than laps >50 (Figure 5D, Wilcoxon rank sum test, Bonferroni corrected for multiple comparisons, with pre-correction threshold of *p* < 0.05).

### VTE at the first and second choice points was correlated

**Figure 6:**
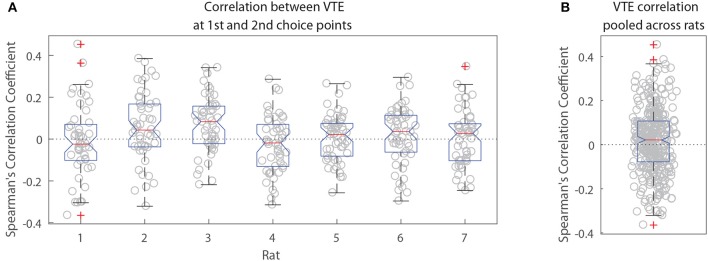
Correlation between VTE at the first and second choice points. **(A)** Correlation coefficients per session for each rat individually. **(B)** Correlation coefficients per session pooled across rats.

The two-step task contains two choice points within a single trial, which enabled us to evaluate how deliberative behavior changed within trial. We found that the amount of VTE at the first and second choice points on a given lap were correlated (Figure 6, the median Spearman's correlation coefficient between LogIdPhi at the first and second choice points within a session was >0, two-sided Wilcoxon signed rank test, *N*_*sessions*_ = 357, *p* = 0.0337, median ρ = 0.0215). Considered individually, two individual rats showed significant positive correlations, while no rats showed significant negative correlations (Figure 6A and Table 3). We also fit a mixed model to VTE at the two choice points, which accounted for rat- and session-specific differences in VTE, and still found a significant positive correlation between the levels of VTE at the two choice points on single laps (Table 4). This suggests that instead of deliberating at each single choice independently, rats may have entered a deliberative mode for entire trials, where then each individual decision within that trial was made using the deliberative system.

**Table 3:** Spearman's correlations between VTE at choice point 1 and choice point 2 for each rat.

**Rat**	**Median *ρ***	***p***
1	−0.0243	0.406
2	0.0430	0.0267
3	0.0834	0.00109
4	−0.0185	0.178
5	0.0216	0.661
6	0.0359	0.198
7	0.0270	0.982

*Shown are the median correlation coefficients (over sessions from that rat) and the p-value of a Two-sided Wilcoxon signed rank test*.

**Table 4:** Mixed Model of the correlation between VTE at the two choice points.

**Mixed model of the correlation between VTE at the two choice points**						
**Parameter**	**2.5%**	**Estimate**	**97.5%**	**t-statistic**	**DF**	***p***
*β*	0.0570	0.0685	0.0801	11.7	26,457	2.65 × 10^−31^
σ_*r*_	0.129	0.225	0.392			
σ_*s*_	0.341	0.369	0.401			
σ_ϵ_	0.896	0.904	0.912			
**Parameter descriptions**						
**Parameter**			**Description**					
*β*			Standardized coefficient					
σ_*r*_			Standard deviation of the per-rat random effect					
σ_*s*_			Standard deviation of the per-session random effect					
σ_ϵ_			Standard deviation of the residual error					

### VTE and path stereotypy changed with the number of choice repeats

**Figure 7:**
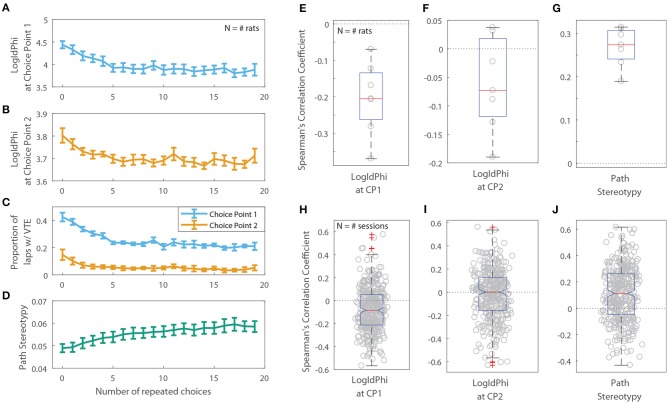
VTE and Path Stereotypy as a function of the number of repeated choices. Raw levels of VTE at the first **(A)** and second **(B)** choice points, the ratio of laps on which rats showed VTE **(C)**, and path stereotypy **(D)** as a function of choice repeats. For **(A–D)**, error bars show mean ± SEM with *N* = 7, the number of rats. **(E,F)** Per-rat correlation coefficients between the number of repeated choices and VTE at the first choice point **(E)**, second choice point **(F)**, and path stereotypy **(G)**. **(H–J)** Per-session correlation coefficients between the number of repeated choices and VTE at the first choice point **(H)**, second choice point **(I)**, and path stereotypy **(J)**.

Previous rodent research has found that animals transition from displaying deliberative behavior to stereotyped behavior over the course of a session, or with experience on a task. If this shift toward stereotyped behavior is due to procedural learning, then a decrease in deliberative behavior and a corresponding increase in stereotyped behavior should also be apparent as a function of the number of repeated choices an animal makes. For the two-step task, we defined a “repeated choice” to be when a rat made the same choice at both the first and second choice points as on the previous lap. We found that VTE at the first choice point was negatively correlated with the number of repeated choices rats made on the two-step task (Figures 7A,E,H; the per-rat median Spearman's correlation coefficient between LogIdPhi at the first choice point and the number of choice repeats was <0, two-sided Wilcoxon signed rank test, *N*_*rats*_ = 7, *p* = 0.0156, median ρ = −0.205). On the other hand, path stereotypy was positively correlated with the number of repeated choices (Figures 7D,G,J; the per-rat median Spearman's correlation coefficient between path stereotypy and the number of choice repeats was >0, two-sided Wilcoxon signed rank test, *N*_*rats*_ = 7 , *p* = 0.0156, median ρ = 0.274). We found no significant correlation between VTE at the second choice point and the number of choice repeats (Figures 7B,C,F,I; the per-rat median Spearman's correlation coefficient between LogIdPhi at the second choice point and the number of choice repeats was not significantly different from 0, two-sided Wilcoxon signed rank test, *N*_*rats*_ = 7 , *p* = 0.156, median ρ = −0.0730).

However, the amount of VTE at the second choice point did change depending on whether the transition on that lap was common or rare. We fit linear mixed models for VTE at the first choice point, for VTE at the second choice point, and for path stereotypy, with transition type (common or rare) on the current and previous laps as fixed variables, and rat and session as random variables. There was a significant increase in the amount of VTE at the second choice point following a rare transition (Table 5B). VTE at the first choice point on the lap following a transition did not significantly differ between common and rare transitions (Table 5A). Path stereotypy on a given lap, however, was significantly decreased when there was a rare transition either on that lap or on the preceding lap (Table 5C).

**Table 5:** Mixed models of **(A)** VTE at the first choice point, **(B)** VTE at the second choice point, and **(C)** and path stereotypy, with transition type on the current lap and previous lap as fixed effects, and rat and session as random effects.

**Parameter**	**2.5%**	**Estimate**	**97.5%**	**t-statistic**	**DF**	***p***
**(A) MIXED MODEL FOR LogIdPhi AT CHOICE POINT 1**						
*β*_0_	3.979	4.168	4.357	43.28	26106	<10^−100^
*T*	−0.01987	0.009811	0.03949	0.6479	26106	0.517
*T*_*P*_	−0.004464	0.02525	0.05496	1.666	26106	0.0958
σ_*r*_	0.1424	0.2476	0.4307			
σ_*s*_	0.3734	0.4049	0.4390			
σ_ϵ_	0.9622	0.9706	0.9790			
**(B) MIXED MODEL FOR LogIdPhi AT CHOICE POINT 2**						
*β*_0_	3.696	3.726	3.756	243.8	26106	<10^−100^
*T*	0.01528	0.02556	0.03584	4.874	26106	1.100 × 10^−06^
*T*_*P*_	−0.009982	0.0003090	0.01060	0.05892	26106	0.9530
σ_*r*_	0.01959	0.03678	0.06906			
σ_*s*_	0.1006	0.1095	0.1191			
σ_ϵ_	0.3334	0.3363	0.3392			
**(C) MIXED MODEL FOR PATH STEREOTYPY**						
*β*_0_	0.04815	0.05263	0.05712	23.00	25965	<10^−100^
*T*	−0.001344	−0.0008540	−0.0003650	−3.420	25965	6.276 × 10^−4^
*T*_*P*_	−0.001006	−0.0005160	−0.00002600	-2.064	25965	0.03900
σ_*r*_	0.0033665	0.0058732	0.010247			
σ_*s*_	0.0095065	0.010264	0.011082			
σ_ϵ_	0.015813	0.015951	0.01609			
**Parameter descriptions**						
**Parameter**			**Description**	
*β*_0_			Fixed intercept			
*T*			Fixed effect of rare transition on current lap			
*T*_*P*_			Fixed effect of rare transition on previous lap			
σ_*r*_			Standard deviation of the per-rat random effect			
σ_*s*_			Standard deviation of the per-session random effect				
σ_ϵ_			Standard deviation of the residual error				

*The 2.5% column indicates the lower bound of the 95% confidence interval, and the 97.5% column indicates the upper bound of the 95% confidence interval. DF, degrees of freedom*.

To determine what may have been driving VTE at the first choice point, we fit a mixed model of VTE at the first choice point, with random effects of rat and session, and with fixed effects of the transition on the previous lap, whether the rat repeated its previous choice, and the delay on the previous lap. We found that VTE at the first choice point was driven by a complex interaction between these three factors (Table 6). Confirming our previous results, there was not a detectable main effect of the transition on the previous lap, and there was a significant negative correlation between VTE at the first choice point and repeated choices. There was also a significant positive correlation between delay on the previous lap and VTE at the first choice point. Several of the interaction terms and the three-way interaction were also significant. Taken together, this suggests that VTE at the first choice point reflects a deliberative process, where the interaction between many task variables are being taken into account, instead of simply being driven by a single task variable such as transition.

**Table 6:** Mixed model of VTE at the first choice point.

**Mixed model for LogIdPhi at choice point 1**						
**Parameter**	**2.5%**	**Estimate**	**97.5%**	***t-*****statistic**	**DF**	***p***
*β*_0_	4.142	4.328	4.514	45.59	26110	<10^−100^
*T*_*P*_	−0.2222	−0.1086	0.005064	−1.873	26110	0.0611
*C*	−0.5217	−0.4608	−0.3999	−14.83	26110	1.60 × 10^−49^
*D*_*P*_	0.002559	0.00567	0.00878	3.573	26110	0.000354
*T*_P_*C	0.08108	0.2126	0.3441	3.168	26110	0.00153
*T*_P_*D_*P*_	−0.0007716	0.005783	0.01234	1.729	26110	0.0838
C*D_*P*_	0.008183	0.0119	0.01562	6.272	26110	3.62 × 10^−10^
*T*_P_*C*D_*P*_	−0.02229	−0.01439	−0.006489	−3.57	26110	0.000358
*σ*_*r*_	0.1358	0.2353	0.4077			
*σ*_*s*_	0.3282	0.3566	0.3874			
*σ*_ϵ_	0.9514	0.9597	0.9678			
**Parameter descriptions**						
**Parameter**			**Description**				
β_0_			Fixed intercept			
*T*_*P*_			Fixed effect of rare transition on previous lap				
*C*			Fixed effect of choice repeat on current lap				
*D*_*P*_			Fixed effect of delay on the previous lap				
*σ*_*r*_			Standard deviation of the per-rat random effect				
*σ*_*s*_			Standard deviation of the per-session random effect				
*σ*_ϵ_			Standard deviation of the residual error				

Transition type on the previous lap, delay on the previous lap, and whether the rat repeated its choice or not are fixed effects, and rat and session are random effects. A*B indicates an interaction term between A and B. The 2.5% column indicates the lower bound of the 95% confidence interval, and the 97.5% column indicates the upper bound of the 95% confidence interval. DF, degrees of freedom.

These results indicate that VTE at the first and second choice points may have been partially driven by different factors. VTE at the first choice point occurred more often when rats had just switched to a new choice pattern and interactions between task variables, but was not detectably affected by the transition on the previous lap alone. On the other hand, VTE at the second choice point occurred more often after an unexpected transition, but was not detectably affected by choice repetitions. We hypothesize that VTE at the first choice point arises more as a result of some deliberative process, which in theory also decreases with the number of repeated choices. Conversely, we hypothesize that VTE at the second choice point, when not being driven by a deliberative mode, arises more as a result of the interruption of a procedural process, which may lead to deliberation, because it is influenced more strongly by unexpected transitions in the middle of a lap than by a change in choice patterns.

## Discussion

We found that rat choices on the two-step task were better explained by a mixture of the model-based and model-free systems than by either system alone. Furthermore, we were able to use the fact that each lap on the two-step task contained multiple decisions to evaluate how VTE changed over the course of a trial, and found that VTE at the two choice points were correlated within lap, suggesting rats likely entered deliberative modes for entire laps. We also observed that VTE at the first choice point was more strongly driven by changes in choice patterns and interactions between task variables, suggesting a deliberative process, while VTE at the second choice point was more strongly driven by unexpected mid-lap changes in state action outcomes, suggesting an interruption in a procedural process.

The correlation between VTE at the two choice points may seem inconsistent with our interpretation that VTE at the second choice point is driven by an interruption of a procedural process. However, we do not believe that VTE at the second choice point is being driven entirely by such interruptions. Rather, we would hypothesize that VTE at the second choice point likely co-occurs with VTE at the first choice point when rats are in a deliberative mode, and that VTE at the second choice point is only primarily driven by rare transitions when rats are in a procedural mode and the unexpected transition interrupts their stereotyped behavior.

Our findings are consistent with previous work in humans which finds that hybrid algorithms are more likely to explain behavior than model-based algorithms alone, and that the weights in these hybrid algorithms favor model-free decision-making (Daw et al., 2011; Voon et al., 2015), though see Simon and Daw (2011) and Gillan et al. (2015). However, some work in rodents on the two-step task finds that rodent choices are primarily, but not necessarily exclusively, model-based, or “planning-driven” (Akam et al., 2013; Miller et al., 2013, 2014, 2017). This discrepancy could have been caused by any of several factors, but we suspect differences in how we implemented the two-step task for rodents was the main contributor.

There were some specific differences between our version of the two-step task and that used by others. Unlike the human version of the two-step task (Daw et al., 2011) and other rodent adaptations (Miller et al., 2017), we used delay to reward delivery as the cost, instead of the probability of reward delivery. We also implemented the full version of the two-step task, with costs which changed according to a random walk, and no second stage choice cue. The more simplified version used in rodents by Miller et al. (2017) had costs which switched between blocks of trials but stayed constant throughout a block, and had a cued second stage choice.

We found that reinforcement learning models were difficult to fit to rat choices on our task. The number of MCMC iterations required to obtain fits whose chains converged was extremely high (see section Methods), and attempting to fit multilevel models (models with rat as a mixed effect) only aggravated this problem. Furthermore, the fit learning rates of our reinforcement learning models were suspiciously low. We suspect that the complexity of our version of the two-step task for rodents, along with the use of delay to reward delivery as the cost, prevented the rats from learning the task well enough to employ solely the model-based system, and so relied also on the model-free system in order to make choices on the task. This may explain why we found that a mix of model-based and model-free strategies best explained rat choices on our task.

We noticed that some rats preferred certain feeders over multiple days, regardless of delay (data not shown). It could be that Pavlovian decision-making or place preferences also played a role in some rats' choices. This might explain in part the relatively low values of the fit second-stage learning rates (see Table 2). In the current analysis, we chose not to model side biases in order to limit our models to the simplest set of model features which were able to capture model-based vs. model-free choices. However, it would be informative in future work to investigate and model the influences of other decision-making systems in addition to only the model-based and model-free systems.

Hierarchical learning, or “chunking” of action sequences, is thought to occur when multiple actions are chained together and are able to be released as a single action. While action chains are usually thought to be driven by a model-free system, some work suggests that model-based systems are capable of initiating action chains which may appear driven by procedural learning (Dezfouli and Balleine, 2012, 2013; Dezfouli et al., 2014). In future work, it would be interesting to investigate if and how the effects of hierarchical learning on the two-step task affect (or are affected by) arbitration between systems.

Our task used the same two physical locations for the four second-stage end states. Although the task included auditory and visual cues, some rats may have confused the two second-stage end states which shared the same location (for example they may have confused E and C, or D and F, see Figure 1B). This may have caused some “bleeding” between the expected values of state-action pairs which led to those states. Any confusion of states in this way would have been an error in situation recognition, and would not necessarily have been occurring in the model-based or model-free systems themselves. Situation recognition is thought to be carried out by a separate system, one not intrinsic to the model-based or model-free systems themselves (Fuhs and Touretzky, 2007; Redish et al., 2007; Gershman et al., 2015). Therefore, any confusion between states would presumably affect both the model-based and model-free systems equally. For this reason we decided not to model any bleeding of state-action values because we were interested only in differences between the model-based and model-free systems.

We adapted the two-step decision task from Daw et al. (2011) for rats in order to study behavioral correlates of model-free and model-based decision-making, but this version of the task can also be used to study neural correlates of model-free and model-based decision-making using electrophysiological techniques in the rodent brain. Representation of state-action pairs and “task-bracketing” in dorsolateral striatum have been hypothesized to initiate action sequences which have been learned procedurally (Jog et al., 1999; Frank, 2011; Regier et al., 2015). On the other hand, model-based neural activity has been observed in a variety of brain areas including hippocampus, ventral striatum, orbitofrontal cortex, prefrontal cortex, and dorsomedial striatum (Johnson and Redish, 2007; van der Meer et al., 2012; Daw and Dayan, 2014; Wikenheiser and Redish, 2015; Brown et al., 2016), and inactivating the dorsal hippocampus in rats impairs model-based decisions (Miller et al., 2017). The current behavioral analysis assumes that either the model-based or model-free system is used to make a decision, but it would be informative to record from the neural structures implicated in procedural learning and those involved in deliberation in rats as they run the two-step task to determine if and how the two systems operate concurrently. Importantly, Akam et al. (2015) suggest that certain model-free strategies can appear to generate model-based choices on the two-step task. Therefore, if these systems may not be able to be conclusively dissociated based purely on choice patterns, it will be important for further work to investigate neural activity in brain areas thought to drive model-based or model-free decision making in order to truly disentangle the contribution of each system.

By adapting for rats a decision task which is made up of multi-choice trials, we were able to investigate how rats used model-free and model-based choice strategies on the task, along with how the transition from deliberation to procedural automation occurs over the course of single trials, and over the course of sequences of repeated choices. We found that a mixture of model-based and model-free choice strategies was more likely to explain rats' choices on this task than either strategy alone. Furthermore, we found that VTE at the two choices within a trial were correlated, which suggests that rats entered deliberative or procedural modes for whole laps. Also, vicarious trial and error at the first choice in a trial corresponded to a complex interaction between task variables and the number of repeated choices, suggesting a deliberative process. Conversely, we found that vicarious trial and error at the second choice in a trial corresponded to unexpected transitions, suggesting it was driven by interruptions in a procedural process which triggered deliberation.

## Methods

### Task

We adapted for rats the human two-stage choice task from Daw et al. (2011). The task consisted of two choice points (“stages”) where subjects were presented with a choice between two options, and then were presented with a second choice between two additional options. The options available at the second stage depended probabilistically on the choice made at the first stage: there were two possible second-stage contexts, each of which was presented 80% of the time after its corresponding 1st-stage decision (a common transition), while the opposite second-stage choice was presented 20% of the time (a rare transition). Upon making a choice at the second stage, subjects were given a reward which corresponded to that second-stage choice. Reward values differed between the four possible second-stage outcomes, and so the objective of the task was to make first- and second-stage decisions which led to the greatest amount of reward.

For rats, we used T-shaped left/right choice points in a spatial maze as the choices. The task consisted of two such choice points encountered serially, where both choices at the first T led to the same physical second choice. Each choice at the second T led to one of two 45-mg food pellet dispensers (MedAssociates, St. Albans, VT) on either side of the maze, at which point rats were required to wait a certain amount of time before they received two food pellets per lap. We used delay to food as a proxy for reward, instead of reward probability as was used in Daw et al. (2011) and Miller et al. (2017). Higher delays corresponded to lower value, and lower delays to higher value. Delays ranged between 1 and 30 s, and changed over the course of the session according to a Gaussian random walk with a standard deviation of 1 s/lap. The decision at the first choice point probabilistically controlled which of the two possible second-stage contexts were encountered. To indicate to the animal which second-stage context they were in, we presented auditory and visual cues after the first choice was made. The auditory cue was a beep pattern unique to each second stage, and the visual cue was white-on-black lines or circles (depending on the second stage) displayed on three Dell S2340M monitors around the second choice point. From the pellet dispensers on either side of the maze, there were return hallways to the start of the maze. There was another pellet dispenser at the start of the maze, where rats received one pellet per lap. Four one-way doors were used to prevent the rats from moving backwards through the maze: one on either side of the first choice-point, and one just before entry into the reward offer zone. Rats were allowed to freely run the task for the duration of sessions which lasted 45 min, and earned their food for the day while running the task (~10–15 g).

### Subjects

Seven male Brown Norway rats aged 6–15 months obtained from Harlan (Bloomington, Indiana) were subjects for the experiment. Before behavioral training, rats were handled for 7 d, then acclimated for 7 d to eat the food pellets delivered during the task (45-mg sucrose pellets, Test Diet), and finally trained to run through the one-way doors on a separate maze for 7 d. Rats were housed on a 12-h light-dark cycle, and behavioral sessions were run at the same time daily. Rats were food restricted to encourage them to run the task, and maintained weight at >80% of their free-feeding weight. Water was always available in their home cage. All experimental and animal care procedures complied with US National Institutes of Health guidelines for animal care and were approved by the Institutional Animal Care and Use Committee at the University of Minnesota.

### Behavioral recording

Animal behavior on the task was captured with a video camera (Cohu, Inc.) placed above the maze. Custom Matlab (MathWorks) software determined animal position from the video on-line; controlled delays, pellet dispensers, and monitors; and recorded animal trajectory through the maze along with task events. Custom Matlab software was written to track animal head positions from video off-line.

### Training

There were three phases of task training, each lasting 8 d. For the first, there was no delay to food delivery, no second-stage auditory or visual cues, and one option was blocked at each choice point, leaving only one possible path through the maze. Choices were blocked such that all four paths through the maze (LL, LR, RL, RR) were sampled equally. Eight pellets were dispensed at the two feeder sites per reward on the first day of training, and the number of pellets decreased by 1 pellet every 2 days for the duration of the training phase. A single pellet per lap was dispensed at the rear feeder site.

For the second training phase, there were still no second-stage auditory or visual cues, and one of the first-stage options was blocked, but both second-stage choices were left open. Delay to food was set randomly between 1 and 10 s on the first day of second phase training, and the maximum delay increased by 2 s/day for the duration of the training phase. The delay values were allowed to change over the course of the session according to the same Gaussian random walk used in the full task (but not allowed to increase above the maximum delay for the day). Four pellets were dispensed at each feeder site for the first 4 days of this training phase, and three pellets for the last 4 days.

The third training phase was 8 d of the full task, with no choices blocked, a maximum delay of 30 s, and two pellets per feeder site.

### Analysis

All analyses except the Bayesian modeling were performed in Matlab (MathWorks). The Bayesian reinforcement learning model fits were performed in Stan (Carpenter et al., 2017) using the Python interface PyStan (Stan Development Team, 2017).

#### Vicarious trial and error (VTE)

To measure VTE for each pass of a rat through the choice point zone, we used LogIdPhi, which captures both how long the rat hesitates at the choice point, and how quickly the rat's head is changing direction. When *x* and *y* are the position of the rat's head,

$$\begin{array}{l} \text{LogIdPhi}=\log\left(\int_{\mathrm{zone entry}}^{\mathrm{zone exit}}|\frac{\delta }{\mathrm{\delta t}}\;\text{atan2}\left(\frac{\mathrm{\delta y}}{\mathrm{\delta t}},\frac{\mathrm{\delta x}}{\mathrm{\delta t}}\right)|\mathrm{\delta t}\right) \end{array}$$

On a very small proportion of choice point passes, we were unable to compute VTE due to a momentary lag in the rat position tracking system. At the first choice point, this occurred on 13 laps (0.049% of laps). At the second choice point, this occurred on 10 laps (0.038% of laps). We excluded these laps from our analysis.

#### Path stereotypy

To measure path stereotypy, we used the inverse of the mean distance between the path on a given lap and all other paths during the same session of the same type (LL, LR, RL, or RR), re-sampled in time. This resulted in a value which was larger when paths were more stereotyped (similar to the average path), and smaller for irregular paths through the maze. When a lap was the only lap of its type in a session, we could not calculate path stereotypy (with no similar paths for which to compute the mean distance), and so we excluded such laps from our analysis. These laps made up a very small proportion of the total data (0.66%).

### Algorithm fits

Each algorithm computed the expected value (or *Q*-value) of taking an action *a*, in any given state, *s*. Our model of the two-step task included only two possible actions in any state (“go left” or “go right”), and only three states: the first choice point (C1, a choice between A and B), and the two possible second choice points (C2, a choice between C and D; and C3, a choice between E and F) see diagram in Figure 1.

The next three subsections explain how each algorithm computes the expected value (or *Q*-value) of taking an action *a*, in any given state, *s*. The section after that (“Computing the Likelihood of Each Algorithm”) describes how the likelihood is computed for each algorithm from that algorithm's *Q*-values. This “likelihood” is the probability that the algorithm, with a given set of values for its parameters, would make the same choices we observed the rats make on the two-step task. The section after that (“Bayesian Algorithm Fitting Using Stan”) describes how the models are fit using these likelihoods. Finally, the section after that (“Algorithm Comparison”) describes how algorithms were compared to determine which one was most likely to explain our data.

#### Model-free algorithm

For the model-free algorithm, we used the SARSA(λ) temporal difference learning algorithm (Rummery and Niranjan, 1994), as was used in Daw et al. (2011). This algorithm learns the expected value (*Q*_*MF*_) of taking a given action *a*, in any given state *s*, by updating the *Q*-values according to the delta rule:

$$\begin{array}{l} Q_{MF}\left(s_{i,t},a_{i,t}\right)=Q_{MF}\left(s_{i,t},a_{i,t}\right)+\alpha_{i}\delta_{i,t} \end{array}$$

where *s*_*i, t*_ is the state on trial *t* at stage *i*, and *a*_*i, t*_ is the action taken in that state on that trial. α_*i*_ is the learning rate for stage *i*. There were only two stages on the two-step task: decisions at the first stage (C1) used α_1_, and decisions at the second stage (C2 or C3, see Figure 1) used α_2_. The reward prediction error, δ_*i, t*_, was the difference between expected and experienced reward on trial *t* at stage *i*:

$$\begin{array}{l} \delta_{i,t}=r_{i,t}+Q_{MF}\left(s_{i+1,t},a_{i+1,t}\right)-Q_{MF}\left(s_{i,t},a_{i,t}\right) \end{array}$$

where *r*_*i, t*_ is the reward experienced at stage *i* of trial *t*. For the first stage reward, we defined *r*_1, *t*_ = 0, because rats did not receive reward between the first and second choice points. For the second stage rewards, we defined the reward as the opposite of the cost:

$$\begin{array}{l} r_{2,t}=maxDelay-d_{2,t} \end{array}$$

where *maxDelay* is the maximum possible delay to food on our task (30 s), and *d*_2, *t*_ is the delay experienced on trial *t* (and explicit delays only occurred after a choice at stage 2). This assumes that rats are aware of the maximum delay, which we believe is a valid assumption, because rats were trained extensively on the task before the experiment began. We also defined a third “virtual” state, where *Q*_*MF*_(*s*_3, *t*_, *a*_3, *t*_) = 0, because there is no further reward in a trial following food delivery. The algorithm updates the first-stage state-action value based on the eligibility trace parameter and second-stage reward prediction error at the end of each trial:

$$\begin{array}{l} Q_{MF}\left(s_{1,t},a_{1,t}\right)=Q_{MF}\left(s_{1,t},a_{1,t}\right)+\alpha_{1}\lambda \delta_{2,t} \end{array}$$

Note that the update for *Q*_*MF*_(*s*_1, *t*_, *a*_1, *t*_) occurs twice per trial: once after the first-stage choice (where the α_1_ learning rate is used), and again after the end of the trial according to the eligibility trace parameter, λ (where a learning rate of (α_1_λ) is used, as in the equation above).

#### Model-based algorithm

The model-based algorithm updates the second-stage state-action values [*Q*(*a*_2, *t*_, *s*_2, *t*_)] in exactly the same way as the model-free system. However, for the first-stage state action values, instead of updating them according to the delta rule, the model-based algorithm takes into account the transition probabilities and the best option at either second stage, and computes the first-stage action values at decision time by:

$$\begin{array}{l} Q_{MB}(s_{A},a_{t})=p(s_{B}|s_{A},a_{t})\;\underset{a^{\prime }\in \{a_{A},a_{B}\}}{\max}Q_{MF}(s_{B},a^{\prime })\;\\ \;+\;p(s_{C}|s_{A},a_{t})\;\underset{a^{\prime }\in \{a_{A},a_{B}\}}{\max}Q_{MF}(s_{C},a^{\prime }) \end{array}$$

where, *s*_*A*_ is the first-stage state, *s*_*B*_ is one of the two second-stage states, *s*_*C*_ is the other second-stage state, and *a*_*t*_ is an action taken at the first stage of trial *t*. *p*(*s*_*X*_|*s*_*Y*_, *a*_*t*_) is the transition probability from state *s*_*Y*_ to *s*_*X*_ after taking action *a*_*t*_ at *s*_*Y*_. Because the rats were trained on the two-step task for over 3 weeks before we started collecting the data to which these models were fit, we assumed the rats had learned the transition probabilities by the end of training, and so our model did not include the learning of the transition probabilities. Therefore, *p*(*s*_*X*_|*s*_*Y*_, *a*_*t*_) was set to either 0.8 for a common transition or 0.2 for a rare transition.

#### Constant-weight hybrid algorithm

This algorithm values actions according to some constant weight between the model-based and model-free algorithm values. Essentially, the constant-weight hybrid algorithm “runs” both the model-free and model-based algorithms simultaneously, and then computes the value (*Q*_*CW*_) of taking some action *a* in some state *s* as the weighted average between the state-action values of the model-free and model-based systems:

$$\begin{array}{l} Q_{CW}\left(s,a\right)=wQ_{MB}\left(s,a\right)+\left(1-w\right)Q_{MF}\left(s,a\right) \end{array}$$

where *w* is a free parameter which determines the weighting between the model-based and model-free systems. If *w* = 1 then the algorithm is purely model-based, and if *w* = 0 then the algorithm is purely model-free. The model-based and model-free algorithms within the constant-weight hybrid algorithm are assumed to share parameters, as in Daw et al. (2011).

#### Computing the likelihood of each algorithm

To transform each algorithm's valuations of different state-action pairs (each algorithm's *Q*-values) into probabilities that the algorithm would make the same choice as the rats did at stage *i* of trial *t* [we denote this probability by *p*(*a*_*i, t*_ = *a*|*s*_*i, t*_)], we used a softmax for each algorithm, in the same way as in Daw et al. (2011):

$$\begin{array}{l} p(a_{i,t}=a|s_{i,t})=\frac{\exp(\beta_{i}[Q(s_{i,t},a)+p\times rep(a)])}{\underset{a^{\prime }}{\sum^{\text{​}}}\exp(\beta_{i}[Q(s_{i,t},a^{\prime })+p\times rep(a^{\prime })])} \end{array}$$

where β_*i*_ is an inverse temperature parameter that controls how stochastic the models' choices are at each choice point, and the sum in the denominator sums over all available actions, *a*′. As *β*_*i*_ → 0, the choices become purely random, and as *β*_*i*_ → ∞, the probability of choosing the action with the largest *Q* value approaches 1. We used independent *β*_*i*_ parameters for each stage of the task, and the *i* index of *β*_*i*_ corresponds to the stage. There were only two stages on the two-step task. Decisions at the first stage (C1) used *β*_1_, and decisions at the second stage (C2 or C3, see Figure 1) used *β*_2_.

The *p* parameter accounts for an inclination to repeat the same action taken on the last lap (*p* > 0), or to switch to the opposite action (*p* < 0), regardless of expected action values. *rep*(*a*) was a function which evaluated to 1 if the rat repeated its action, that is, performed action *a* at that stage on the previous lap (stage *i*, trial *t* − 1), and 0 if it chose a different action. Therefore, if the *p* parameter was positive, the algorithm was more likely to repeat the previous choice, and if it was negative, the algorithm was more likely to switch (choose the opposite choice from the previous trial).

We initialized all *Q*-values to the mean reward value at the beginning of each session. The log probability of observing rat choices across all *N*_*s*_ sessions given an algorithm is then:

$$\begin{array}{l} \log\left(p\left(\text{data}|\theta \right)\right)=\overset{N_{d}}{\underset{d=1}{\sum }}\overset{N_{t}}{\underset{t=1}{\sum }}\overset{N_{i}}{\underset{i=1}{\sum }}\log\left(p\left(a_{i,t}=a|s_{i,t}\right)\right) \end{array}$$

where *θ* is the set of all parameters for a given algorithm, *N*_*i*_ is the number of choice stages in each trial *t* (for our task this is always 2: the first choice point, C1, and the second choice point, C2 or C3, see Figure 1), *N*_*t*_ is the number of trials in a given session (or “day”) *d*, and *N*_*d*_ is the total number of sessions across all rats.

#### Bayesian algorithm fitting using stan

We used Markov chain Monte Carlo (MCMC) in Stan (Carpenter et al., 2017), and the Python programming language interface to Stan, PyStan (Stan Development Team, 2017), to generate model parameter posterior distributions so that we could perform model comparison and inference of the parameter values. Stan is a platform for Bayesian statistical modeling (http://mc-stan.org), in which models can be written using a simple modeling language, and Stan performs MCMC sampling resulting in model and parameter posterior probabilities. This allowed us to perform Bayesian inference as to the values of model parameters, and model comparison using DIC scores.

In Table 2, for each algorithm we report the DIC score, the median of the MCMC samples for all parameters, and 95% confidence intervals. Each algorithm was fit in PyStan with 5 chains per algorithm, and 10,000 iterations per chain (5,000 warm-up and 5,000 sampling). Chains which took longer than 96 h to run were aborted and re-started. We used pooled (non-hierarchical) models, such that the same parameter was used for each rat.

We used vaguely informative priors for the Bayesian fits in Stan. Across all models, the priors used were:

**Table:** 

**Parameter**	**Prior**
α_1_	Beta distribution with α = 1.2, *β* = 1.2
α_2_	Beta distribution with α = 1.2, *β* = 1.2
λ	Beta distribution with α = 1.2, *β* = 1.2
*β*_1_	Exponential distribution with λ = 0.5
*β*_2_	Exponential distribution with λ = 0.5
*p*	Normal distribution with μ = 0, σ = 10
*w*	Beta distribution with α = 1.2, *β* = 1.2

#### Algorithm comparison using dic

The three algorithms which were fit to rat behavior did not all have the same number of parameters:

**Table:** 

**Algorithm**	**Number of parameters**	**List of parameters**
Model-free	6	α_1_, α_2_, λ, *β*_1_, *β*_2_, and *p*
Model-based	4	α_2_, *β*_1_, *β*_2_, and *p*
Constant-weight	7	α_1_, α_2_, λ, *β*_1_, *β*_2_, *p*, and *w*

Using naive model comparison methods, like comparing model likelihoods, could cause models with more parameters to be deemed more likely due to overfitting. In order to compare the probability of models which have different numbers of parameters, we used Deviance Information Criterion (DIC) (Spiegelhalter et al., 2002). DIC allows a more fair comparison of models with different numbers of parameters by penalizing models which have a higher effective number of parameters. It is also well-suited for use with models whose posterior distributions have been computed via MCMC, which is the method we used. We compute the DIC score by:

$$\begin{array}{l} DIC=D\left(\bar{\theta }\right)+2p_{D} \end{array}$$

where the effective number of parameters (*p*_*D*_) is computed by:

$$\begin{array}{l} p_{D}=\bar{D}-D\left(\bar{\theta }\right) \end{array}$$

$\bar{D}$ is the average of the deviance, *D*(*θ*), over all the MCMC samples of *θ*:

$$\begin{array}{l} \bar{D}=\frac{1}{N_{samples}}\overset{N_{samples}}{\underset{i=1}{\sum }}D\left(\theta_{i}\right) \end{array}$$

$D\left(\bar{\theta }\right)$ is the deviance evaluated at the average of the MCMC samples of *θ*:

$$\begin{array}{l} D\left(\bar{\theta }\right)=D\left(\frac{1}{N_{samples}}\overset{N_{samples}}{\underset{i=1}{\sum }}\theta_{i}\right) \end{array}$$

The deviance is computed by:

$$\begin{array}{l} D\left(\theta \right)=-2\log\left(p\left(\text{data}|\theta \right)\right) \end{array}$$

where log(*p*(data|*θ*_*i*_)) is the algorithm likelihood, as computed above (in section Computing the Likelihood of Each Algorithm), given parameters *θ* for a MCMC sample. The deviance is technically *D*(*θ*) = −2log(*p*(data|*θ*))+*C*, but *C* is a constant which cancels out when comparing different models. Algorithms are compared based on their DIC scores, where models with lower DIC scores are more likely to explain the data.

### Model-based and model-free simulations

We simulated model-based and model-free agents on the two-step task for Figures 2, 3. We used the same models which were fit to rat behavior (above) to simulate agent choice on the task, and used the same task parameters which were used for the rats. Simulated sessions lasted 74 laps (the average length of rat sessions).

The data used for the “chance” line in Figure 2 was generated by 10,000 simulated sessions of a model-free agent with learning rates set to 0 (α_1_, α_2_ = 0, *β*_1_, *β*_2_ = 3, *p*, λ = 0). We used an agent with learning rates set to 0 because this yielded an agent whose choice probabilities were 50% for any choice on the model of our task, since the *Q*-values were all initialized to the same value.

The model-free and model-based simulations in Figures 3A,B were generated by 10,000 simulated sessions of model-free or model-based agents. Parameters used were α_1_, α_2_ = 0.5, *β*_1_, *β*_2_ = 3, *p* = 0.3, λ = 0.

### Mixed model of VTE at the two choice points

To determine if levels of VTE were correlated between the two choice points, we fit a mixed model to LogIdPhi at the first and second choice points. Specifically, the model tried to predict LogIdPhi at the second choice point from LogIdPhi at the first choice point on that same lap. These models included subject and session as random effects; that is, they allowed levels of VTE to vary across subjects and sessions, but not in a totally independent way. Our model included a fixed intercept, a fixed effect of transition type on the current lap, a fixed effect of transition type on the previous lap, a per-subject random effect, and a per-session random effect.

$$\begin{array}{l} \begin{array}{ccc} {\text{zLogIdPhi}}_{2,i} & = & \beta_{0}+\beta_{VTE}\times {\text{zLogIdPhi}}_{1,i}+R_{r}+S_{s}+\epsilon \\ R & \sim & N\left(0,\sigma_{r}\right)\\ S & \sim & N\left(0,\sigma_{s}\right)\\ \epsilon & \sim & N\left(0,\sigma_{e}\right) \end{array} \end{array}$$

where

- *zLogIdPhi*_2, *i*_ is the z-scored LogIdPhi value at the second choice point on lap *i*,
- *zLogIdPhi*_1, *i*_ is the z-scored LogIdPhi value at the first choice point on lap *i*,
- *β*_0_ is the fixed intercept of the model (baseline LogIdPhi),
- *β*_*VTE*_ is the standardized coefficient (a parameter which captures the relationship between the amount of VTE at the two choice points),
- *R*_*r*_ is rat *r*'s random effect (or adjustment coefficient), which accounts for the possibility that some rats have different baseline levels of LogIdPhi,
- *S*_*S*_ is session *s*'s random effect, which accounts for the possibility that rats have different baseline levels of LogIdPhi on different sessions,
- *σ*_*r*_ and *σ*_*s*_ are the standard deviations of per-rat (*R*) and per-session (*S*) random effects, respectively,
- *σ*_*e*_ is the standard deviation of the error, and
- N(μ,σ) represents a normal distribution centered at μ with standard deviation σ.

*β*_0_ was not reported in Table 4, because the inputs to the model were z-scored, and so the intercept was, of course, not significantly different from 0.

### Mixed models of VTE and path stereotypy with transition type

In order to determine if VTE and path stereotypy changed depending on whether there was a rare transition on the current or previous lap, we fit mixed models to VTE and path stereotypy. These models included subject and session as random effects; that is, they allowed levels of VTE or path stereotypy to vary across subjects and sessions, but not in a totally independent way. Our model included a fixed intercept, a fixed effect of transition type on the current lap, a fixed effect of transition type on the previous lap, a per-subject random effect, and a per-session random effect.

$$\begin{array}{l} \begin{array}{ccc} Y_{i} & = & \beta_{0}+Tt_{i}+T_{P}t_{i-1}+R_{r}+S_{s}+\epsilon \\ R & \sim & N\left(0,\sigma_{r}\right)\\ S & \sim & N\left(0,\sigma_{s}\right)\\ \epsilon & \sim & N\left(0,\sigma_{e}\right) \end{array} \end{array}$$

where

- *Y*_*i*_ is the LogIdPhi value at the first choice point on lap *i* (or the LogIdPhi value at the second choice point on lap *i* for the second choice point model, or the path stereotypy value on lap *i* for the path stereotypy model),
- *β*_0_ is the intercept of the model (baseline LogIdPhi or path stereotypy value),
- *T* is the parameter capturing the fixed effect of rare transitions on the current lap,
- *t*_*i*_ is an indicator variable which is 0 when there was a common transition on lap *i*, and 1 when there was a rare transition on lap *i*,
- *T*_*P*_ is the parameter capturing the fixed effect of a rare transition on the previous lap,
- *t*_*i*−1_ is an indicator variable which is 0 when there was a common transition on lap *i*−1, and 1 when there was a rare transition on lap *i*−1,
- *R*_*r*_ is rat *r*'s random effect (or adjustment coefficient), which accounts for the possibility that some rats have different baseline levels of LogIdPhi or path stereotypy,
- *S*_*S*_ is session *s*'s random effect, which accounts for the possibility that rats have different baseline LogIdPhi or path stereotypy values on different sessions,
- *σ*_*r*_ and *σ*_*s*_ are the standard deviations of per-rat (*R*) and per-session (*S*) random effects, respectively,
- *σ*_*e*_ is the standard deviation of the error, and
- N(μ,σ) represents a normal distribution centered at *μ* with standard deviation σ.

Laps which were the first in a session were not used in this analysis, as the transition type of the previous (non-existent) lap was undefined. The degrees of freedom in the mixed model for path stereotypy were different from the degrees of freedom in the mixed models for VTE because on some laps path stereotypy could not be calculated (when a lap was the only lap of that type in a session, see the Path Stereotypy section above). Also the degrees of freedom in the mixed models for VTE are different here than for the mixed model used between VTE at the two choice points, because this model does not include laps which were the first in a session (see above).

### Mixed model of VTE at the first choice point

In order to determine what was contributing to VTE at the first choice point, we fit a mixed model to VTE at the first choice point. This model included subject and session as random effects, a fixed intercept, a fixed effect of transition type on the previous lap, a fixed effect of delay experienced on the previous lap, and a fixed effect of choice repetition (whether the previous choice was repeated or not).

$$\begin{array}{l} \begin{array}{ccc} Y_{i} & = & \beta_{0}+T_{P}t_{i-1}+D_{P}d_{i-1}+Cc_{i}+R_{r}+S_{s}+\epsilon \\ R & \sim & N\left(0,\sigma_{r}\right)\\ S & \sim & N\left(0,\sigma_{s}\right)\\ \epsilon & \sim & N\left(0,\sigma_{e}\right) \end{array} \end{array}$$

where

- *Y*_*i*_ is the LogIdPhi value at the first choice point on lap *i*
- *β*_0_ is the intercept of the model (baseline LogIdPhi value),
- *T*_*P*_ is the parameter capturing the fixed effect of a rare transition on the previous lap,
- *t*_*i*−1_ is an indicator variable which is 0 when there was a common transition on lap *i*−1, and 1 when there was a rare transition on lap *i*−1,
- *D*_*P*_ is the parameter capturing the fixed effect of the delay on the previous lap,
- *d*_*i*−1_ is the delay in seconds on lap *i*−1,
- *C* is the parameter capturing the fixed effect of choice repetition,
- *c*_*i*_ in an indicator variable which is 0 when the rat did not repeat its choice on lap *i*, and 1 when it did,
- *R*_*r*_ is rat *r*'s random effect (or adjustment coefficient), which accounts for the possibility that some rats have different baseline levels of LogIdPhi or path stereotypy,
- *S*_*S*_ is session *s*'s random effect, which accounts for the possibility that rats have different baseline LogIdPhi or path stereotypy values on different sessions,
- *σ*_*r*_ and *σ*_*s*_ are the standard deviations of per-rat (*R*) and per-session (*S*) random effects, respectively,
- *σ*_*e*_ is the standard deviation of the error, and
- N(μ,σ) represents a normal distribution centered at μ with standard deviation *σ*.

## Author contributions

BH and ADR conceived and designed the experiment and analyses, and wrote the manuscript. BH collected and analyzed the data.

### Conflict of interest statement

The authors declare that the research was conducted in the absence of any commercial or financial relationships that could be construed as a potential conflict of interest.
