# Corrigendum: Deliberation and Procedural Automation on a Two-Step Task for Rats

**DOI:** 10.3389/fnint.2018.00040

**Published:** 2018-09-25

**Authors:** Brendan M. Hasz, A. David Redish

**Affiliations:** ^1^Graduate Program in Neuroscience, University of Minnesota Twin Cities, Minneapolis, MN, United States; ^2^Department of Neuroscience, University of Minnesota Twin Cities, Minneapolis, MN, United States

**Keywords:** decision-making, reinforcement learning, model-based, model-free, vicarious trial and error, path stereotypy

In the original article, there was an error. In the abstract, the sentence “While VTE at the first choice point increased with the number of repeated choices, VTE at the second choice point did not, and only increased after unexpected transitions within the task.” should read “While VTE at the first choice point decreased with the number of repeated choices, VTE at the second choice point did not, and only increased after unexpected transitions within the task.” The corrected abstract should read:

“Current theories suggest that decision-making arises from multiple, competing action-selection systems. Rodent studies dissociate deliberation and procedural behavior, and find a transition from procedural to deliberative behavior with experience. However, it remains unknown how this transition from deliberative to procedural control evolves within single trials, or within blocks of repeated choices. We adapted for rats a two-step task which has been used to dissociate model-based from model-free decisions in humans. We found that a mixture of model-based and model-free algorithms was more likely to explain rat choice strategies on the task than either model-based or model-free algorithms alone. This task contained two choices per trial, which provides a more complex and non-discrete per-trial choice structure. This task structure enabled us to evaluate how deliberative and procedural behavior evolved within-trial and within blocks of repeated choice sequences. We found that vicarious trial and error (VTE), a behavioral correlate of deliberation in rodents, was correlated between the two choice points on a given lap. We also found that behavioral stereotypy, a correlate of procedural automation, increased with the number of repeated choices. While VTE at the first choice point decreased with the number of repeated choices, VTE at the second choice point did not, and only increased after unexpected transitions within the task. This suggests that deliberation at the beginning of trials may correspond to changes in choice patterns, while mid-trial deliberation may correspond to an interruption of a procedural process.”

The authors apologize for this error and state that this does not change the scientific conclusions of the article in any way.

The original article has been updated.

## Conflict of interest statement

The authors declare that the research was conducted in the absence of any commercial or financial relationships that could be construed as a potential conflict of interest.

